# Thrombocytosis in COVID-19 patients without myeloproliferative neoplasms is associated with better prognosis but higher rate of venous thromboembolism

**DOI:** 10.1038/s41408-021-00585-2

**Published:** 2021-11-29

**Authors:** Marko Lucijanic, Ivan Krecak, Ena Soric, Martina Sedinic, Anica Sabljic, Lovorka Derek, Ozren Jaksic, Rajko Kusec

**Affiliations:** 1 Hematology Department, University Hospital Dubrava, Zagreb, Croatia; 2 University of Zagreb School of Medicine, Zagreb, Croatia; 3 Internal Medicine Department, General hospital Sibenik, Sibenik, Croatia; 4 Clinical Department for Laboratory Diagnostics, University Hospital Dubrava, Zagreb, Croatia

**Keywords:** Infectious diseases, Risk factors, Haematological diseases

**Dear Editor,**

Platelet functions are well known to surpass thrombosis and hemostasis and platelets play an important role in inflammatory and profibrotic mechanisms. Thrombocytopoiesis is traditionally considered to occur in the bone marrow. However, thrombocytopoiesis was shown to occur in the lungs as well where circulating megakaryocytes release platelets and are responsible for up to 50% of total platelet production [1]. The specific role of platelets in the lungs is not fully elucidated. Platelets are the source of cytokines that were shown to support lung regeneration and embryonic development (SDF1, TGF-β1, IGF1), and fetal lung megakaryocytes in comparison to bone marrow megakaryocytes were shown to exhibit higher transcription of these factors [2]. Thrombocytopenia is recognized as a negative prognostic factor in COVID-19 patients [3]. Nevertheless, there are no data on the prognostic significance of elevated platelet count that can be encountered in COVID-19 patients, besides indirect conclusions based on reported outcomes in chronic myeloproliferative neoplasm (MPN) patients [4, 5].

We have retrospectively analyzed a cohort of 5876 consecutively hospitalized COVID-19 patients who were admitted to our tertiary-level institution (University Hospital Dubrava, Zagreb, Croatia) in the period from March 2020 to June 2021, had baseline complete blood count available, and did not have known prior or subsequent MPN. COVID-19 severity on admission was defined by the World-Health-Organization guidelines as mild, moderate, severe, and critical. Clinical and laboratory data used in the study are part of the hospital registry project and were obtained by analysis of written and electronic medical records. A total of 88.8% of patients received LMWH thromboprophylaxis in dose intensity per the assessment of treating physicians, 74.6% received steroids and 14.9% received remdesivir. Acetylsalicylic acid use corresponded to baseline indication for chronic use (18%). Platelet count and mean platelet volume (MPV) were obtained by Advia 2120i counter (Siemens-Medical-Solutions-Diagnostics-Pte-Ltd., Swords, Ireland). The normality of the distribution of numerical variables was tested using the Shapiro–Wilk test. Since the majority of numerical variables did not have normal distribution they were presented as the median and interquartile range (IQR) and were analyzed using the non-parametrical tests (the Mann–Whitney-*U* test and the Kruskal–Wallis-analysis-of-variance with the post-hoc test by Conover). Categorical variables were presented as frequencies and percentages and were analyzed using the *Χ*^2^-test. Time to event analyses for survival were based on the Kaplan–Meier method. The log-rank test and the Cox regression were used for univariate and multivariate survival analyses. Follow-up was considered from the time of hospital admission up to 114 days, including the post-discharge period (to correspond to the follow-up time of the longest hospitalized patient). Clinical outcomes of interest (death from any cause, mechanical ventilation (MV), bacteriemia, venous and arterial thromboses proven by objective imaging and laboratory methods, major bleeding defined by the International-Society-on-Thrombosis-and-Hemostasis criteria) were considered during the hospitalization period and were evaluated as binary variables. The logistic regression was used for univariate and multivariate analyses for the prediction of non-survival outcomes. All analyses were done using the MedCalc statistical software, version 20.014 (MedCalc-Software-Ltd, Ostend, Belgium; 2021).

Among 5876 patients, there were 3299 (56.1%) males. The median age was 72 years, IQR (62-81), and median Charlson comorbidity index (CCI) was 4 points, IQR (3–6). COVID-19 severity on admission was mild in 528 (9%), moderate in 281 (4.8%), severe in 4168 (70.9%) and critical in 899 (15.3%) patients. The median platelet count was 221 × 10^9^/L, IQR (164–297), and median MPV was 9 fL, IQR (8.4–9.7). A total of 1120 (19.1%) patients presented with thrombocytopenia (platelets <150 × 10^9^/L). Three-hundred-and-six (5.2%) patients had platelets <100 × 10^9^/L and 55 (0.9%) had platelets <50 × 10^9^/L. A total of 270 (4.6%) patients had thrombocytosis (platelets ≥450 × 10^9^/L). Sixty-three (1.1%) patients had platelets ≥600 × 10^9^/L.

Patients’ characteristics stratified by platelet count on admission are shown in Table 1. As shown, patients with thrombocytosis were significantly more likely to be younger, of female sex and to have lower comorbidity burden (CCI) than patients with thrombocytopenia (*P* < 0.05 for all comparisons) and of similar age, sex and comorbidity burden as patients with the normal number of platelets (differences not significant (n.s.)). Regarding specific comorbidities, arterial hypertension, diabetes mellitus, hyperlipoproteinemia, and active smoking were similarly distributed between subgroups of patients with different platelet count (n.s.) whereas patients with thrombocytosis were significantly less likely to be obese and more likely to have active malignancy (mostly gynecologic malignancy) than patients with normal platelet count, and less likely to have liver cirrhosis, chronic kidney disease or active anti-cancer therapy than patients with thrombocytopenia (*P* < 0.05 for all comparisons). COVID-19 severity on admission and presence of pneumonia did not significantly differ between subgroups of patients with different platelet count. However, the duration of COVID-19 symptoms significantly gradually increased in subgroups of patients presenting with increasing platelet count, with patients with thrombocytopenia presenting earlier and patients with thrombocytosis presenting later during the disease course in comparison to patients with normal platelet count (*P* < 0.05 for all comparisons).

**Table 1:** Patients’ characteristics on admission stratified according to the baseline platelet count.

	Plts < 150 × 10^9^/L (1)	Plts 150–449 × 10^9^/L (2)	Plts ≥ 450 × 10^9^/L (3)	*P* value
Number of pts	1120 (19.1%)	4486 (76.3%)	270 (4.6%)	–
Age (years)	74 (64.5–82)	72 (62–81)	71 (61–80)	*P* < 0.001^a^ (1 vs. 2; 2 vs. 3)^a^
*Sex*				
Male	714 (63.7%)	2438 (54.3%)	147 (54.4%)	*P* < 0.001^a^ (1 vs. 2; 1 vs. 3)^a^
Female	406 (36.2%)	2048 (45.7%)	123 (45.6%)	
Time from symptoms onset (days)	4 (1–8)	6 (2–10)	7 (2–12)	*P* < 0.001^a^ (1 vs. 2; 1 vs. 3; 2 vs. 3)^a^
Pneumonia	1018 (90.9%)	4044 (90.1%)	239 (88.5%)	*P* = 0.476
*COVID-19 severity*				
Mild	93 (8.3%)	407 (9.1%)	28 (10.4%)	*P* = 0.219
Moderate	57 (5.1%)	204 (4.5%)	20 (7.4%)	
Severe	803 (71.7%)	3190 (71.1%)	175 (64.8%)	
Critical	167 (14.9%)	685 (15.3%)	47 (17.4%)	
ECOG status on admission	3 (2–4)	2 (1–3)	2 (1–4)	*P* < 0.001^a^ (1 vs. 2; 1 vs. 3)^a^
MEWS symptom severity	2 (1–4)	2 (1–4)	2 (1–4)	*P* = 0.598
Charlson comorbidity index	5 (3–7)	4 (2–6)	4 (2–6)	*P* < 0.001^a^ (1 vs. 2; 1 vs. 3)^a^
Arterial hypertension	772 (68.9%)	3074 (76.5%)	171 (63.3%)	*P* = 0.185
Diabetes mellitus	346 (30.9%)	1319 (29.4%)	77 (28.5%)	*P* = 0.569
Hyperliproteinemia	237 (21.2%)	1037 (23.1%)	52 (19.3%)	*P* = 0.155
Obesity	280 (25%)	1383 (30.8%)	62 (23%)	*P* < 0.001^a^ (1 vs. 2; 2 vs. 3)^a^
Active smoking	57 (5.1%)	255 (5.7%)	17 (6.3%)	*P* = 0.650
Active malignancy	156 (13.9%)	372 (8.3%)	32 (11.9%)	*P* < 0.001^a^ (1 vs. 2; 2 vs. 3)^a^
Malignancy type^b^				P < 0.001^a^
Lung cancer	8 (5.1%)	54 (14.5%)	5 (15.6%)	
Breast cancer	6 (3.8%)	21 (5.6%)	1 (3.1%)	
Colon cancer	10 (6.4%)	53 (14.2%)	5 (15.6%)	
Stomach cancer	4 (2.6%)	10 (2.7%)	2 (6.2%)	
Hematologic malignancy	82 (52.6%)	75 (20.2%)	3 (9.4%)	
Gynecologic malignancy	5 (3.2%)	16 (4.3%)	5 (15.6%)	
Other	41 (26.3%)	143 (38.4%)	11 (34.4%)	
Active anticancer therapy	62 (5.5%)	76 (1.7%)	7 (2.6%)	*P* < 0.001^a^ (1 vs. 2; 1 vs. 3)^a^
Liver cirrhosis	40 (3.6%)	31 (0.7%)	1 (0.4%)	*P* < 0.001^a^ (1 vs. 2; 1 vs. 3)^a^
Chronic kidney disease	191 (17.1%)	445 (9.9%)	24 (8.9%)	*P* < 0.001^a^ (1 vs. 2; 1 vs. 3)^a^
Prior aspirin use	188 (16.8%)	819 (18.3%)	59 (21.9%)	*P* = 0.142
Prior anticoagulant therapy	287 (25.6%)	1023 (22.8%)	60 (22.2%)	*P* = 0.123
WBC ×10^9^/L	5.8 (4.1–8.2)	8.3 (6.1–11.5)	11.2 (8.4–14.8)	*P* < 0.001^a^ (1 vs. 2; 1 vs. 3; 2 vs. 3)^a^
Abs. neutrophils ×10^9^/L	4.4 (2.9–6.6)	6.7 (4.6–9.7)	8.8 (6.3–12.6)	*P* < 0.001^a^ (1 vs. 2; 1 vs. 3; 2 vs. 3)^a^
Abs. lymphocytes ×10^9^/L	0.7 (0.5–1)	0.8 (0.6–1.2)	1 (0.7–1.6)	*P* < 0.001^a^ (1 vs. 2; 1 vs. 3; 2 vs. 3)^a^
Abs. monocytes ×10^9^/L	0.3 (0.2–0.5)	0.43 (0.3–0.6)	0.59 (0.4–0.8)	*P* < 0.001^a^ (1 vs. 2; 1 vs. 3; 2 vs. 3)^a^
Abs. eosinophils ×10^9^/L	0 (0–0.02)	0.01 (0–0.03)	0.02 (0–0.1)	*P* < 0.001^a^ (1 vs. 2; 1 vs. 3; 2 vs. 3)^a^
Abs. basophils x10^9^/L	0.01 (0–0.03)	0.02 (0–0.05)	0.04 (0.02–0.08)	*P* < 0.001^a^ (1 vs. 2; 1 vs. 3; 2 vs. 3)^a^
Hemoglobin g/L	127 (110–141)	129 (116–142)	120 (105–132)	*P* < 0.001^a^ (1 vs. 2; 1 vs. 3; 2 vs. 3) ^a^
RDW %	14.4 (13.5–15.7)	13.9 (13.3–14.9)	14.1 (13.4–15.3)	*P* < 0.001^a^ (1 vs. 2; 1 vs. 3; 2 vs. 3)^a^
Platelets ×10^9^/L	119 (96–136)	240 (195–302)	530 (481–596)	*P* < 0.001^a^ (1 vs. 2; 1 vs. 3; 2 vs. 3)^a^
MPV fL	9.5 (8.8–10.4)	8.9 (8.3–9.6)	8.5 (8.1–9)	*P* < 0.001^a^ (1 vs. 2; 1 vs. 3; 2 vs. 3)^a^
Ferritin µg/L	858 (416–1745)	756 (408–1375)	745 (387–1300)	*P* = 0.003^a^ (1 vs. 2; 1 vs. 3)^a^
d-dimers mg/L FEU	1.34 (0.7–3.4)	1.29 (0.69–3.2)	2 (1–4.2)	*P* < 0.001^a^ (1 vs. 3; 2 vs. 3)^a^
LDH U/L	346 (251–482)	364 (264–490)	343 (272–461)	*P* = 0.073
CRP mg/L	80.7 (38.7–135.4)	89.3 (39.7–151.6)	91.4 (40.3–150.7)	*P* = 0.012^a^ (1 vs. 2)^a^
IL-6 pg/mL	57.9 (27.9–120.2)	43.1 (14.4–97.8)	17.5 (5.6–128.3)	*P* < 0.001^a^ (1 vs. 2; 1 vs. 3)^a^

Results are presented as median and interquartile range for continuous variables which are compared between groups using the Kruskal–Wallis ANOVA and post-hoc test by Conover. Categorical variables are presented as frequencies and percentages and are compared between groups using the *Χ*^2^ test.

*Plts* platelets, *pts* patients, *ECOG* Eastern Cooperative Oncology Group, *MEWS* Modified Early Warning Score, *WBC* white blood cells, *Abs.* absolute, *RDW* red blood cell distribution width, *MPV* mean platelet volume, *LDH* lactate dehydrogenase, *CRP* C-reactive protein, *IL* interleukin.

^a^Statistically significant at level *P* < 0.05.

^b^Percentage of patients with active malignancy is shown.

Patients with higher MPV were more likely to be older, of the male sex, to have higher CCI and more severe COVID-19 on admission (*P* < 0.05 for all comparisons). MPV was not significantly associated with the duration of symptoms prior to admission. MPV was significantly negatively correlated with platelet count (Rho = −0.28; *P* < 0.001).

During hospitalization a total of 1991 (33.9%) patients died, 1033 (17.4%) required MV, 609 (10.4%) experienced bacteriemia, 362 (6.2%) venous thromboembolism (VTE), 326 (5.5%) arterial thrombosis and 184 (3.1%) major bleeding. Considering VTE, a total of 135 (2.3%) patients experienced deep venous thrombosis (DVT) and 260 (4.4%) experienced pulmonary embolism (PE). Considering arterial thrombosis events, a total of 102 (1.7%) patients experienced myocardial infarction, 139 (2.4%) cerebrovascular insult, 66 (1.1%) peripheral artery thrombosis and 18 (0.3%) mesenterial thrombosis. Univariate associations of platelet count with clinical outcomes are summarized in Supplementary Table S1 and Fig. 1A. Patients with thrombocytosis experienced significantly better overall survival in comparison to both patients with normal platelet count ((hazard ratio) HR 0.75; *P* = 0.016) and thrombocytopenia (HR 0.47; *P* < 0.001) with in-hospital mortality rates of 24.1%, 31.5%, and 45.6%, respectively. Gradually improving overall survival could be observed in patients with higher platelets, even with further subdivision of patients with low, normal, and elevated platelet count as shown in Fig. 1B (*P* < 0.001 for difference and *P* < 0.001 for trend; platelets stratified to <50, 50–99, 100–149, 150–299, 300–449, 450–599 and ≥600 × 10^9^/L). Similarly, patients with thrombocytosis were less likely to experience respiratory deterioration and require MV in comparison to patients with normal and low platelet count (*P* < 0.001 for difference and *P* < 0.001 for trend; MV rates of 11.5%, 17.1% and 21.2%, respectively), less likely to experience bacterial sepsis (*P* < 0.001 for difference and *P* < 0.001 for trend; bacteriemia rates of 7.4%, 9.6%, and 14%, respectively) and less likely to experience major bleeding (*P* = 0.031 for difference and *P* = 0.020 for trend; major bleeding rates of 0.7%, 3.1%, and 3.8%, respectively). Patients with thrombocytosis had a higher propensity to develop VTE in comparison to patients with normal and low platelet count (*P* = 0.002 for difference and *P* < 0.001 for trend; VTE rates of 9.6%, 6.4%, 4.3%, respectively) whereas there was no significant difference in the occurrence of arterial thrombosis (n.s. for difference and n.s. for trend; arterial thrombosis rates of 4.4%, 5.6%, 5.7%, respectively). Further considering thrombotic events, platelet count was significantly higher in both patients experiencing PE vs. other (median 248 vs. 220 × 10^9^/L; *P* < 0.001) and DVT vs. other (median 250 vs. 221 × 10^9^/L; *P* = 0.020) whereas there was no significant association of platelet count with arterial thrombosis subtypes (n.s). Neither presence of active malignancy, active treatment, nor specific malignancy subtype showed statistically significant associations with VTE occurrence (n.s.). Univariate associations of MPV with clinical outcomes are summarized in Supplementary Table S1 and Supplementary Fig. 1A. Higher MPV stratified to quartiles was significantly associated with worse overall survival (Supplementary Fig. S1B; *P* < 0.001 for difference and *P* < 0.001 for trend; in-hospital mortality rates of 27.5%, 28.4%, 35.4%, and 45.6% for 1st–4th MPV quartile, respectively) and patients belonging to 4th in comparison to 1st MPV quartile also had a higher risk for MV (OR 1.39; *P* < 0.001) and arterial thrombosis (OR 1.52; *P* = 0.009), whereas there were no significant differences in venous thrombosis, bacteriemia and major bleeding between patients belonging to different MPV quartiles (n.s).

**Fig. 1:**
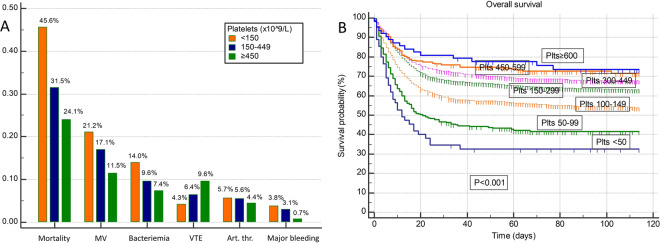
Platelet count associations with clinical outcomes in hospitalized COVID-19 patients. **A** Associations of platelet count (×10^9^/L) on admission with in-hospital mortality, mechanical ventilation (MV), bacteriemia, venous thromboembolism (VTE), arterial thrombosis and major bleeding. **B** Overall survival stratified by platelet count on admission.

Above mentioned associations of platelets and MPV with clinical outcomes (with the exception of MPV and MV) remained statistically significant in the multivariate analyses adjusted for platelets, MPV, age, sex, COVID-19 severity, and CCI. Independent prognostic factors and associated adjusted hazard/odds ratios are shown in Supplementary Table S2.

There are several important points that we would like to emphasize. A much smaller proportion of COVID-19 patients presents with thrombocytosis (5%) than with thrombocytopenia (19%). Platelet count seems to be dominantly affected by specific comorbidities and duration of COVID-19 as opposed to its severity on presentation. Patients with higher platelets were more likely to have a longer duration of COVID-19 symptoms, stronger stimulation of myelopoiesis, and different, probably more mature inflammatory profile (higher WBC subsets, CRP and D-dimers but lower ferritin and IL-6). Elevated platelets were accompanied by a more favorable course of the disease, with less need for MV and less deaths. Even among patients with low, normal, and elevated platelets, further subgroups of patients could be stratified over whom a linear trend of improved survival with increasing platelets exists. Mechanisms behind these observations are elusive but could be attributable to the protective role of platelets for the lung parenchyma and more effective viral clearance by a higher number of platelets. Higher platelets were also associated with a lower rate of bacterial sepsis highlighting their important role in the immune system. It is of special interest that the frequency of VTE was significantly higher among COVID-19 patients with higher platelet count. These findings highly resemble phenomena reported in MPN patients with COVID-19 where patients with essential thrombocythemia (ET) who presented with higher platelet count in comparison to other MPN subsets [5] were reported to have a higher risk for VTE [5] but favorable disease course [4]. Hence, our data suggest that these observations might not be MPN-specific and could represent biological phenomena present in patients with non-clonally elevated platelet count as well. Arterial thromboses had no association with platelet count but were significantly associated with higher platelet volume. Similar to platelets, MPV reflected the need for MV and survival in univariate analyses. Associations of lower platelets and higher MPV with shorter survival seem to be independent of each other and of older age, male sex, more severe COVID-19, and higher comorbidity burden. Nevertheless, an association of MPV with MV was diminished after adjustments for platelet count and severity of COVID-19, probably due to baseline correlations with these parameters. Higher MPV might represent an influx of younger platelets due to their increased consumption and a higher degree of platelet activation due to inflammatory COVID-19 milieu.

Our findings are limited by single-center experience, retrospective study design, and no data on intensity and frequency of use of anticoagulant and antiplatelet therapies at the specific time of thrombotic events. Although we excluded patients with known or subsequently proven MPN, some patients could still have unrecognized MPN but their number is likely to be very low considering MPN incidence in an overall population. The main strengths of our study are a large sample size and adequate statistical power to perform presented analyses. These results provide new insights into the biology of thrombosis and hemostasis in COVID-19 patients where abnormalities in hematologic findings are common and difficult to interpret directly. The reactive thrombocytosis in COVID-19 patients seems to have important clinical correlations and deserves further research.

## Supplementary information

Supplementary Tables and Figures
