# Supplementary material for: Thrombocytosis in COVID-19 patients without myeloproliferative neoplasms is associated with better prognosis but higher rate of venous thromboembolism
Source: Blood Cancer J. 2021 Nov 29;11(11):189. doi: 10.1038/s41408-021-00585-2 (PMC8629336; doi:10.1038/s41408-021-00585-2)

**Supplementary Table S1:** Univariate regression analyses for overall survival, mechanical ventilation, bacteriemia, venous and arterial thrombosis and major bleeding.

| **UNIVARIATE ASSOCIATIONS** | **Overall survival** | **Mechanical ventilation** | **Bacteriemia** | **Venous thrombosis** | **Arterial thrombosis** | **Major bleeding** |
| --- | --- | --- | --- | --- | --- | --- |
| **Plts**  norm vs low | 0.62 (0.56-0.69)  **P<0.001 *** | 0.76 (0.65-0.9)  **P=0.001 *** | 0.65 (0.54-0.79)  **P<0.001 *** | 1.53 (1.12-2.09)  **P=0.008 *** | 0.97 (0.73-1.29)  P=0.854 | 0.8 (0.56-1.13)  P=0.371 |
| **Plts**  high vs low | 0.47 (0.37-0.59)  **P<0.001 *** | 0.48 (0.32-0.72)  **P<0.001 *** | 0.49 (0.3-0.79) **P=0.004 *** | 2.38 (1.45-3.91)  **P<0.001 *** | 0.76 (0.41-1.44)  P=0.411 | 0.19 (0.05-0.77)  **P=0.021 *** |
| **MPV**  2nd vs 1st quartile | 1.05 (0.89-1.22)  P=0.577 | 0.92 (0.83-1.22)  P=0.924 | 1.11 (0.87-1.41)  P=0.385 | 0.89 (0.66-1.19)  P=0.418 | 1.24 (0.89-1.72)  P=0.191 | 0.69 (0.45-1.07)  P=0.100 |
| **MPV**  3rd vs 1st quartile | 1.44 (1.23-1.68)  **P<0.001 *** | 1.19 (0.99-1.44)  P=0.071 | 1.21 (0.96-1.54)  P=0.103 | 0.89 (0.66-1.19)  P=0.425 | 1.34 (0.97-1.86)  P=0.075 | 0.97 (0.66-1.45)  P=0.918 |
| **MPV**  4th vs 1st quartile | 2.21 (1.89-2.57)  **P<0.001 *** | 1.39 (1.15-1.67)  **P<0.001 *** | 1.2 (0.95-1.53); P=0.128 | 0.87 (0.64-1.17)  P=0.341 | 1.52 (1.11-2.09)  **P=0.009 *** | 1.02 (0.68-1.51)  P=0.932 |
| **Age**  years | 1.06 (1.06-1.07)  **P<0.001 *** | 0.99 (0.99-1.0)  P=0.260 | 0.99 (0.98-0.99)  **P=0.025 *** | 0.99 (0.98-1.0)  P=0.132 | 1.02 (1.01-1.03)  **P<0.001 *** | 1.01 (0.99-1.02)  P=0.184 |
| **Male sex** | 1.05 (0.97-1.14)  P=0.254 | 1.76 (1.53-2.03)  **P<0.001 *** | 1.67 (1.4-1.99) **P<0.001 *** | 0.82 (0.66-1.01)  P=0.065 | 1.0 (0.8-1.25)  P=0.987 | 1.17 (0.87-1.58)  P=0.295 |
| **COVID-19 severity** | 2.4 (2.23-2.58)  **P<0.001 *** | 3.94 (3.44-4.51)  **P<0.001 *** | 2.16 (1.87-2.5)  **P<0.001 *** | 1.51 (1.29-1.79)  **P<0.001 *** | 0.86 (0.75-0.99)  **P=0.039 *** | 1.21 (0.99-1.49)  P=0.068 |
| **Charlson comorbidity index** | 1.19 (1.18-1.21)  **P<0.001 *** | 1.01 (0.98-1.04)  P=0.454 | 0.99 (0.97-1.03)  P=0.909 | 0.94 (0.9-0.98)  **P=0.006 *** | 1.16 (1.12-1.2)  **P<0.001 *** | 1.09 (1.04-1.15)  **P<0.001 *** |

*statistically significant at level P<0.05 / Results are presented as hazard ratios with associated 95% confidence intervals for analysis of overall survival (the Cox regression analysis was used) and as odds ratios with associated 95% confidence intervals for analysis of other outcomes during hospitalization (the logistic regression was used). COVID-19 severity and Charlson comorbidity index are analyzed as ordinal variables. / Abbreviations: Plts=platelets; MPV=mean platelet volume.

**Supplementary Table S2:** Multivariate regression analyses for overall survival, mechanical ventilation, bacteriemia, venous and arterial thrombosis and major bleeding.

| **MULTIVARIATE ASSOCIATIONS** | **Overall survival** | **Mechanical ventilation** | **Bacteriemia** | **Venous thrombosis** | **Arterial thrombosis** | **Major bleeding** |
| --- | --- | --- | --- | --- | --- | --- |
| **Plts**  norm vs low | 0.71 (0.64-0.78)  **P<0.001 *** | 0.8 (0.67-0.96)  **P=0.016 *** | 0.67 (0.54-0.82)  **P<0.001 *** | 1.46 (1.06-2)  **P=0.022 *** | 1.15 (0.86-1.55)  P=0.336 | 0.85 (0.59-1.22)  P=0.371 |
| **Plts**  high vs low | 0.61 (0.48-0.79)  **P<0.001 *** | 0.48 (0.31-0.74)  **P<0.001 *** | 0.52 (0.32-0.85); **P=0.009 *** | 2.31 (1.38-3.87)  **P=0.001 *** | 0.93 (0.48-1.77)  P=0.829 | 0.19 (0.05-0.81)  **P=0.025 *** |
| **MPV**  2nd vs 1st quartile | 0.92 (0.81-1.04)  P=0.199 | 0.92 (0.75-1.13)  P=0.436 | 1.03 (0.81-1.31) P=0.803 | 0.88 (0.65-1.17)  P=0.382 | 1.27 (0.91-1.76)  P=0.157 | 0.66 (0.43-1.02)  P=0.065 |
| **MPV**  3rd vs 1st quartile | 1.02 (0.9-1.16)  P=0.716 | 1.06 (0.87-1.3)  P=0.545 | 1.09 (0.86-1.38)  P=0.482 | 0.93 (0.69-1.26)  P=0.669 | 1.29 (0.93-1.79)  P=0.129 | 0.87 (0.58-1.39)  P=0.497 |
| **MPV**  4th vs 1st quartile | 1.26 (1.12-1.42)  **P<0.001 *** | 1.11 (0.9-1.36)  P=0.331 | 0.99 (0.77-1.28) P=0.988 | 0.99 (0.72-1.36)  P=0.971 | 1.42 (1.02-1.98)  **P=0.039 *** | 0.83 (0.55-1.25)  P=0.374 |
| **Age**  years | 1.04 (1.03-1.05)  **P<0.001 *** | 0.99 (0.98-0.99)  **P=0.008 *** | 0.98 (0.97-0.99) **P=0.001 *** | 0.99 (0.98-1.01)  P=0.523 | 1.0 (0.99-1.01)  P=0.722 | 0.99 (0.98-1.01)  P=0.423 |
| **Male sex** | 1.18 (1.09-1.29)  **P<0.001 *** | 1.58 (1.36-1.84)  **P<0.001 *** | 1.51 (1.26-1.81) **P<0.001 *** | 0.78 (0.63-0.98)  **P=0.033 *** | 1.08 (0.86-1.36)  P=0.520 | 1.1 (0.82-1.5)  P=0.515 |
| **COVID-19 severity** | 2.56 (2.37-2.77)  **P<0.001 *** | 3.89 (3.39-4.47)  **P<0.001 *** | 4.85 (3.13-7.51) **P<0.001 *** | 2.27 (1.5-3.4)  **P<0.001 *** | 0.59 (0.44-0.79)  **P<0.001 *** | 1.22 (0.98-1.52)  P=0.067 |
| **Charlson comorbidity index** | 1.11 (1.10-1.13)  **P<0.001 *** | 1.03 (0.99-1.07)  P=0.054 | 1.03 (0.99-1.07) P=0.107 | 0.95 (0.9-1.01)  P=0.101 | 1.14 (1.09-1.19)  **P<0.001 *** | 1.1 (1.04-1.17)  **P=0.001 *** |

*statistically significant at level P<0.05 / Results are presented as hazard ratios with associated 95% confidence intervals for analysis of overall survival (the Cox regression analysis was used) and as odds ratios with associated 95% confidence intervals for analysis of other outcomes during hospitalization (the logistic regression was used). COVID-19 severity and Charlson comorbidity index are analyzed as ordinal variables. / Abbreviations: Plts=platelets; MPV=mean platelet volume.

**Supplementary Figure S1:** **A)** Associations of mean platelet volume (MPV) quartiles on admission with in-hospital mortality, mechanical ventilation (MV), bacteriemia, venous thromboembolism (VTE), arterial thrombosis and major bleeding. **B)** Overall survival stratified by MPV quartiles.


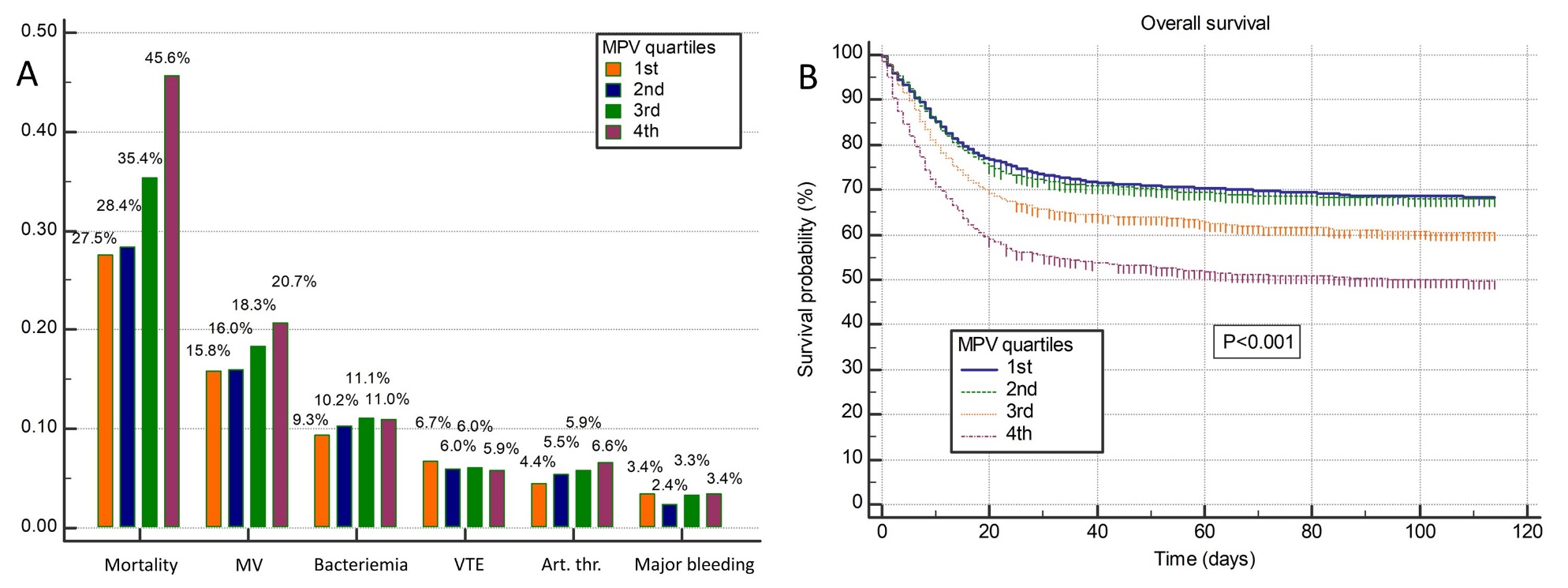

Supplement: Supplementary file 1 — Supplementary Tables and Figures [file 41408_2021_585_MOESM1_ESM.docx]
